# The Dangers of Fiscal Decentralisation in Healthcare: A Response to the Recent Commentaries

**DOI:** 10.34172/ijhpm.2023.8266

**Published:** 2023-09-20

**Authors:** Arianna Rotulo, Christina Paraskevopoulou, Elias Kondilis

**Affiliations:** ^1^Department of Sustainable Health, Campus Fryslân, University of Groningen, Leeuwarden, The Netherlands; ^2^School of Social Sciences, Panteion University of Social and Political Sciences, Attika, Greece; ^3^Faculty of Health Sciences, School of Medicine, Aristotle University of Thessaloniki, Thessaloniki, Greece

 Decentralisation has always been a key element of health systems strengthening; even back to 1978, the World Health Organization (WHO) Alma Ata Declaration clearly stated that the provision of fair, equal, and accessible healthcare should be decentralised and rooted in community-based approaches.^1^ However, decentralisation is not a uniform group of policies. On the contrary, it consists of a heterogeneous set of reforms aiming to the transfer of administrative, political and/or economic power from central governments to subnational authorities.^2^ In this sense decentralisation is not a synonym to fiscal decentralisation, nor the former necessarily entails the latter.

 Fiscal decentralisation is a specific type of decentralisation that occurs when the responsibility to generate, distribute, and spend revenues shifts from the central government to local authorities.^2,3^ That is, when local authorities are financially free of any central regulatory constraint in the pooling and redistribution of resources. This can happen through the introduction of earmarked local taxation and user fees, or through hospital’s autonomization.^3^ Under this type of decentralisation, providers and regional authorities can compete against each other, in an open market environment, on the offer of different *bundles* of public services for a certain taxation level.^4^ In the academic and policy debate the terms “decentralisation” and “fiscal decentralisation” are often used interchangeably,^3^ a fact that inhibits mutual understanding and adds further ambiguity in the relevant policy discussion.

 Fiscal decentralisation (that is, revenue and expenditure decentralisation, and not decentralisation in general) by definition requires the fragmentation of the national pooling system into multiple regional and municipal pools for the financing of health and other public services.^2,3^ This shift from national to local pools is expected to increase efficiency, under the assumptions that local authorities are more accountable to local communities and thus can better meet differing demands and preferences across jurisdictions.^5,6^ On the other hand, pooling of finances is a foundational principle of healthcare financing. Its primary aim is to distribute the financial risk evenly across the population, preventing any single individual from shouldering the whole burden of healthcare expenses, when in need. Under certain circumstances, a funding pool has the capacity to redistribute funds, enabling cross-subsidization among high- and low-risk individuals and income groups.^7^ In financing pooling, size matters: the larger the pool the higher the cross-subsidization.^8^ This basic principle makes national, centralised pools an indispensable financing policy for equalization and universality.^7^ On the contrary, fragmented-local pools weaken the redistributive capacity, as revenues are collected and used within a population sub-group, and limit the cross-subsidisation towards vulnerable and low-income groups at the national level.^7^ This fragmentation of a central pool into several local ones, is intrinsic to fiscal decentralisation, making it a regressive policy option in essence.

 Fiscal decentralisation has also the potential of increasing cross-regional disparities.^3^ In a fiscally decentralised system, sub-national governments engage in a “territorial competition” for the attraction of private or public investments, local revenues, government subsidies, and workforce.^9^ Richer regions with more developed infrastructure, more mature administrative capacity, more and better educated workforce, larger tax bases, higher influence, and preferential treatment from central governments have an obvious advantage over their less developed competitors.^9^ Accordingly, poorer regions with weaker governance structures, lower influence to the central government, and smaller tax bases, face the risk of losing the “territorial race” before it has even begun.^9,10^ Under fiscal decentralisation, the central government’s role in redistributing income and wealth from rich to poor regions, is also significantly weakened.^3^ For all these reasons it is argued that fiscal decentralisation can exacerbate pre-existing inter-regional inequalities, and lead to the concentration of resources to more developed and rich regions.^3,9,10^

 Our study has shown that in Italy between 2001-2017, fiscal decentralisation was related to a decrease in the availability, accessibility, and utilisation of healthcare services, with this negative effect being stronger for public healthcare services (relative to private ones) and more prominent in regions with lower fiscal capacity.^11^ These findings signal that 30 years of fiscal decentralisation implementation in Italy has perpetuated or even exacerbated the pre-existing, cross-regional healthcare inequalities in the country. The theories on the relationship of fiscal decentralisation and regional disparities offer a useful and meaningful interpretation of the empirical phenomena that our study has observed in the case of Italy. Similar empirical observations have also been made in other countries under fiscal decentralisation,^12^ supporting the theoretical concerns regarding the negative impact of fiscal decentralisation on spatial, healthcare inequalities.

 While our study aligns with the theoretical expectations that fiscal decentralisation negatively influences the capacities of public healthcare systems and perpetuates geographical disparities,^11^ the COVID-19 pandemic proved to be a real-life testing ground for the preparedness of fiscally decentralised healthcare systems. Emerging evidence suggests that fiscal decentralisation policies significantly impaired the preparedness and responsiveness of these health systems, in some cases to the point of requiring swift re-centralisation.^13-15^

 Decentralisation is a key element of any healthcare reform that aims to strengthen local healthcare systems and make them more responsive to local health and healthcare needs. In this sense the question in policy debate is not whether to decentralise in general or not, but what functions to decentralise and how.^3^ Under certain circumstances, decentralising administrative, political, and/or healthcare expenditure powers to subnational authorities can increase local effectiveness. On the contrary decentralising healthcare revenue powers to subnational localities entails too many risks and dangers as theory, pre-pandemic empirical evidence, and the COVID-19 experience in fiscally decentralised health systems actually suggest.

 Even in capitalism, redistribution of income and wealth has historically been (under welfarism) and should remain a responsibility of the central, rather than the local, state.^3^ Centralised pooling of resources and centralised resource allocation to regions might not be sufficient (depending on tax progressivity and resource allocation equalizers) but are necessary conditions for any meaningful attempt to redistribute income and wealth among individuals and across jurisdictions.^3^ Accordingly, rejecting fiscal decentralisation might not be a sufficient option to heal inequities and healthcare deficiencies, but it is a necessary condition for any attempt towards that direction.

## Ethical issues

 Not applicable.

## Competing interests

 Authors declare that they have no competing interests.
